# No Effect of Added Sugars in Soft Drink Compared With Sugars in Fruit on Cardiometabolic Risk Factors: Results From a 4-Week, Randomized Controlled Trial

**DOI:** 10.3389/fnut.2021.636275

**Published:** 2021-06-30

**Authors:** Lisa Te Morenga, Simonette R. Mallard, Fabiane B. Ormerod

**Affiliations:** ^1^Department of Human Nutrition, University of Otago, Dunedin, New Zealand; ^2^Riddet Centre of Research Excellence, University of Otago, Dunedin, New Zealand; ^3^Edgar Diabetes and Obesity Research, University of Otago, Dunedin, New Zealand; ^4^School of Health, VIC University of Wellington, Wellington, New Zealand

**Keywords:** fructose, fruit, sugar, cardiometabolic risk, sugar-sweetened soft drink, beverage, dietary intervention

## Abstract

High intakes of added sugar from soft drinks are associated with negative health outcomes such as the increased risk of gout and type 2 diabetes, weight gain and cardiovascular disease. Fruits are naturally high in sugars but their effect on cardiometabolic risk remains unknown. We examined the effect on cardiometabolic risk factors of consuming natural sugars from fruit or added sugars from sugar-sweetened soft drinks in overweight adults. Forty-eight healthy, overweight (BMI ≥ 28 kg/m^2^) men (*n* = 21) and women (*n* = 20) were randomized to either a fruit (*n* = 19) or sugar-sweetened soft drink (*n* = 22) intervention for 4 weeks. The fruit group received 6 items of fresh and dried fruit per day and the sugar-sweetened soft drink group received 955 ml of sugar-sweetened soft drink per day. The interventions were matched for both energy (fruit: 1,800 kJ/d; soft drink: 1,767 kJ/d) and fructose content (fruit: 51.8 g/d; soft drink: 51.7 g/d). The soft drink intervention provided 101 g total sugars, which was all added sugar and the fruit intervention provided 97 g total sugars, which were all natural sugars. Dietary intakes were otherwise *ad libitum*. Despite being asked to consume additional sugar (up to 1,800 additional kJ/d), there were no changes in weight, blood pressure or other cardiometabolic risk factors, except by uric acid, in any of the intervention groups. In conclusion, our findings do not provide any evidence that short-term regular intake of added sugars is linked to higher cardiometabolic risks, with exception of uric acid in overweight men. Public health interventions to prevent obesity and related diseases should focus on the quality of the whole diet rather than only focusing on reducing sugary drinks or sugar intakes.

## Introduction

Increased consumption of added or free sugars is associated with increased risk of obesity and related diseases worldwide (1–3). The evidence linking sugar intakes with obesity-related diseases relates largely to the provision of extra calories with causes weight. The World health organization (WHO) recommends that free sugars (all monosaccharides and disaccharides added to foods by the manufacturer, cook or consumer, plus the sugars that are naturally present in honey, syrups and fruit juices) are limited to <10% of daily energy intake (4). This recommendation is mostly based on findings related to dental caries given relatively limited evidence of increased risk of cardiovascular disease or other negative health outcomes. There has been considerable interest in whether the associations between fructose and negative health outcomes are specifically linked with high intakes of fructose (1, 5–7). High fructose intake has indeed been linked with hyperuricemia (8, 9), risk of gout (5), but has not been found to be associated with risk for type 2 diabetes (10). However, fructose is infrequently consumed in the absence of equal amounts of glucose and therefore reduction of free or added sugars tends to be the target of public health interventions (11).

Sugars in the human diet are mostly presented as glucose, fructose, galactose, lactose, and sucrose (12). Sucrose, commonly consumed as table sugar, is a disaccharide constituted by equal parts of fructose and glucose (12). Fructose-containing sweeteners, such as high fructose corn syrup are presented in different forms but contain fructose and glucose in relatively similar proportions to sucrose (13). Added sugars are most commonly consumed globally in the form of sucrose or high fructose syrups which are commonly used to sweeten soft drinks, desserts and bakery items, confectionary, and other processed foods (11, 14).

Fruit is also an important source of dietary sugars glucose and sucrose and particularly, fructose. Chemically the sugars in fruit and added sugars are indistinguishable and the ratio of glucose to fructose equivalents is similar (1:1) (12). This has led to concerns in some quarters that fruit should also be limited. The strongest evidence for limiting fruit comes from studies which have examined the associations between fruit intake, and gout and hyperuricaemia. Generally, observational studies of varied design have indicated a reduced risk of incident gout (5) or experiencing gout attacks (15) with higher fruit consumption. Contrary to these findings, in an analysis of the Health Professionals Follow-up Study (*n* = 46,393 men), higher fruit juice and fruit intakes were associated with an *increased* risk of incident gout after a 12 years follow-up (5). Acute feeding studies in humans involving fruit or fruit juice have also produced mixed results, with an immediate rise in serum uric acid seen after consuming apples (16) or apple juice (17); a lowered plasma urate level after cherry consumption, and no effect of grapes, strawberries or kiwifruit on plasma urate (18). Finally, in a 6 week weight reduction trial, energy-restricted diets providing either a relatively high intake of fructose from fruit (50–70 g/d) or a low amount of fructose (<10–20 g/d) led to significantly lowered serum uric acid concentrations, although no difference was seen between the diets and the reductions in serum uric acid could have been explained by the weight achieved in both interventions (19). However, fruit is high in micronutrients, antioxidants and dietary fiber, and has a relatively low energy density and fruit intake has been associated with a reduced risk of several chronic diseases (9) as well as better glycemic control in people with type 2 diabetes (20). Clearly there is a need for research to clarify whether the sugars naturally found in fruits have similar effects on disease risks as added sugars.

In the present study, we sought to compare the effect of consuming sugars from either whole fruit or sugar sweetened soft drink (soft drink) on serum cardiometabolic risk factors, over 4 weeks, matched for both energy, fructose and total sugars in addition to an *ad libitum* diet. We hypothesized that due to its more favorable nutritional properties, sugars in fruit would have a more favorable effect on cardiometabolic risk factors than sugars from sugar-sweetened soft drinks.

## Materials and Methods

### Subjects

In September 2012, overweight men and women were recruited using a local newspaper advertisement and University of Otago, Dunedin, New Zealand email lists. Two-hundred and sixty-seven (267) respondents were assessed via an online survey or telephone interview, 48 of whom appeared to meet inclusion criteria: body mass index (BMI) ≥ 28 kg/m^2^; aged between 20 and 75 years; no established diabetes, liver or kidney disease, gout or a history of other major chronic illnesses; no diagnosed mental disorders; not currently taking medications affecting blood pressure, blood lipids, blood glucose or mood/mental state; not currently pregnant or breastfeeding; no intolerance to study fruit or fructose; able to remain in Dunedin for the duration of the intervention period; and willing to consume either fruit or soft drink for 4 weeks. Of the 48 respondents invited to attend a screening visit to confirm their eligibility and obtain written informed consent, seven did not meet inclusion criteria. A total of 41 participants were randomized to consume either fruit (*n* = 19) or soft drink (*n* = 22). Following randomization, but prior to receiving their allocated beverage, three participants withdrew from the study. One participant in the fruit group moved away from Dunedin midway through the intervention and was lost to follow-up, resulting in a final total of 37 participants (*n* = 18 fruit; *n* = 19 soft drink) completing the study by December 2012. This study was approved by University of Otago Human Ethics Committee (Ref: 12/197). The trial was registered with the Australian New Zealand Clinical Trials Registry: ACTRN12612000874819; http://www.anzctr.org.au.

### Experimental Protocol

Computer-generated block randomization was stratified by sex (to account for potential differences uric acid in response to treatment), performed before recruitment, and concealed from researchers in sealed, numbered envelopes. After establishing a participant's eligibility at the screening visit, and obtaining written informed consent, the next envelope was opened and the participant's group allocation revealed. Due to the nature of the study the researcher responsible for delivering the interventions, and participants, could not be blinded to group allocation.

Approximately 1–2 weeks after their initial screening visit, participants attended a baseline visit at the Department of Human Nutrition Clinic, University of Otago, Dunedin, New Zealand. Between screening and baseline, and during the final week of the intervention, participants completed a 3-day weighed diet record, recorded on non-consecutive days and including a weekend day. Electronic scales were provided along with written instructions, and a trained researcher verbally explained how to complete the diet records. The researcher was available by email or telephone to answer questions that arose during completion of the diet record. Dietary intakes of macronutrients, fructose; vitamin C; potassium and dietary fiber were determined using Kaiculator dietary assessment software (University of Otago, Dunedin, NZ), which uses the New Zealand food composition database (21). Participants were informed of their group allocation at baseline. Those randomized to fruit were provided with one Cavendish banana (128 g), three Braeburn or Jazz apples (149 g each) and two 14 g boxes of Sunmaid seedless raisins per day. Those assigned to soft drinks were provided with one 355 mL can and one 600 mL bottle of sugar (sucrose) sweetened soft drink (either Coca-Cola or Sprite) per day. The interventions were matched as closely as possible for energy (fruit: 1,800 kJ/d; soft drink: 1,767 kJ/d), fructose (fruit: 51.8 g/d; soft drink: 51.7 g/d), and totals sugars (fruit: 97 g/d soft drink: 101 g/d) content and all participants were instructed to consume their usual diet as normal. Participants collected fruit and soft drinks weekly, and were asked to record daily consumption in a log booklet, and to return empty beverage containers and any unconsumed fruit/beverages on a weekly basis.

Participants attended a clinic at baseline and at the end of study after an overnight (10–12 h) fast. Anthropometric measurements were made by one trained researcher, during which participants wore light clothing and no shoes. Weight to the nearest 0.1 kg, body fat percentage to the nearest 0.1% and BMI to the nearest 0.1 kg/m^2^ were measured in duplicate using a calibrated Tanita Wedderburn bioimpedance analyzer. Height was measured using a Seca fixed stadiometer, and waist circumference was measured underneath clothing with a non-stretching anthropometric tape according to the International Society for the Advancement of Kinanthropometry protocols (22). Height and waist circumference were measured to the nearest 0.1 cm in duplicate, or in triplicate if measurements differed by >0.5 cm or >1%, respectively. Seated blood pressure was measured after a 5 min rest in triplicate using an Omron digital blood pressure monitor in mm Hg. Fasting blood samples (8 mL) were drawn by a research nurse using ethylenediaminetetraacetic acid (EDTA)-treated vacutainers. Blood samples were kept at <4°C for ~1 h, then centrifuged at 1,650 g for 15 min. Plasma samples were then frozen in polyethylene cryovials at −80°C until analysis.

### Laboratory Analysis

Plasma insulin was measured using a specific electrochemiluminescence immunoassay for the Elecsys analyzer (Roche Diagnostics, Mannheim, Germany), with a coefficient of variation (CV) of 1.1%. Total cholesterol (CV: 1.0%), triglyceride (CV: 0.9%), glucose (CV: 0.8%), and plasma uric acid (CV: 0.9%) concentrations were measured enzymatically with kits and calibrators supplied by Roche Diagnostics on a Cobas Mira analyzer (Roche Diagnostics). High-density lipoprotein (HDL; CV: 1.5%) was measured in the supernatant after precipitation of apolipoprotein B-containing lipoproteins with phosphotungstate/magnesium chloride solution (23). Low-density lipoprotein (LDL) was calculated using the Friedewald equation (24). High sensitivity C-reactive protein (CRP) was measured using a latex-enhanced immunoturbidimetric method (Roche Diagnostics) with a CV of 6.4%.

### Statistical Analysis

The primary outcome measure was serum uric acid and insulin sensitivity measured by the McAuley Index (25). Using an estimated change in mean plasma uric acid of 50 μmol/L (SD of 75 μmol/L) and a correlation between repeated measures of 0.75 (26), it was estimated that 15 participants per group would be required for analysis of covariance (ANCOVA) with one baseline and one follow-up measurement, at 80% power and alpha = 0.05. To allow for attrition, *n* = 40 was the overall recruitment goal.

Change in body composition and clinical measures from baseline to week 4 were compared within treatment groups using Student's *t*-tests. Data were checked for normality and equal variance, with non-normal data compared using Mann–Whitney tests, and data with unequal variance compared using Welch's *t*-tests. The effect of treatment on body composition and clinical measures was analyzed by ANCOVA with baseline values as a covariate. Other participant characteristics likely to affect outcomes (baseline BMI and age) and a potential interaction effect (sex by group) were included as covariates individually, and those with a *P* < 0.25 were retained in the final model. All analyses were conducted using Stata 11.1 (Stata Corporation 2010, College Station, Texas, United States), and a two-sided 0.05 level of significance was used in all cases.

## Results

Table 1 summarizes baseline characteristics of participants randomized to treatment. Thirty-seven participants completed the study, 19 in the fruit group, and 18 in the soft drink group. Nine women completed each intervention. Participants had a mean age of 33.2 years and 54% were obese with mean BMI of 31.4 kg/m^2^.

**Table 1:** Baseline demographic and clinical measures of participants randomized to fruit or soft drink^a^.

	**Fruit (*n* = 19)**	**Soft drink (*n* = 22)**
Female, *n* (%)	9 (47.4)	11 (50.0)
Age, years	34.7 (12.5)	33.2 (12.8)
Weight, kg	91.0 (21.4)	94.5 (15.2)
Height, cm	170.8 (9.4)	170.8 (9.9)
BMI, kg/m^2^	31.0 (5.3)	32.2 (3.4)
Waist circumference, cm	97.3 (17.2)	99.4 (11.4)
Body fat, %	34.4 (8.4)	35.7 (7.6)
**Self-reported physical activity**, ***n*** **(%)**		
Inactive	3 (15.8)	4 (20.0)
Moderately active	6 (31.6)	6 (30.0)
Active	10 (52.6)	10 (50.0)
Systolic blood pressure, mm Hg	122 (15)	120 (13)
Diastolic blood pressure, mm Hg	70 (11)	69 (9)
Triglycerides, mmol/L	1.29 (0.65)	1.19 (0.45)
LDL-cholesterol, mmol/L	2.93 (0.97)	3.19 (0.89)
HDL-cholesterol, mmol/L	1.26 (0.22)	1.32 (0.37)
Total cholesterol, mmol/L	4.78 (1.10)	5.05 (0.97)
Plasma insulin, mIU/ml	12.67 (10.32)	11.61 (4.70)
Plasma glucose, mmol/L	5.44 (0.60)	5.29 (0.37)
Plasma uric acid, μmol/L	334.2 (66.5)	385.5 (79.7)
Insulin sensitivity index^b^	7.23 (2.03)	7.02 (1.31)

a *Data are means (SD) unless otherwise indicated; soft drink, sugar-sweetened soft drink*.

b *McAuley Insulin sensitivity index: Mffm/I = exp[2.63 – 0.28 ln(insulin) – 0.31 ln(TAG)]*.

There were no significant differences in total energy, carbohydrate, total sugars and glucose intakes between treatments (Table 2). However, sucrose intake was higher in the soft drink group, and fructose intake was higher in the fruit group amongst women only. The fruit group also consumed significantly more dietary fiber during treatment. On average participants in the fruit group consumed 92% of the fruit provided (5.5 items per day) and participants in the soft drink group consumed 94% of the beverages provided (900 ml per day).

**Table 2:** Change in dietary intake from baseline to week 4 and difference between treatments.

	**Fruit**		**Soft drink**		**Overall difference between the treatments^a^**	
	**Baseline (mean, SD)**	**Change (mean, SD)**	**Baseline (mean, SD)**	**Change (mean, SD)**	**Difference (Mean, 95%CI)**	***P*-value for difference**
**Energy (kJ)**						
Males	12,409	−879 (6,639)	12,316	218 (3,592)	−1,031 (−3,899, 1,837)	0.467
Females	8,178	930 (1,461)	8,364	891 (2,723)	−93 (−2,881, 2,694)	0.946
**Carbohydrate (g)**						
Males	307	41 (148)	357	14 (162)	−11 (−89, 65)	0.754
Females	227	48 (42)	245	36 (88)	−2 (−76, 72)	0.959
**Total sugars (g)**						
Males	120	52 (80)	153	64 (63)	−40 (−83, 3)	0.068
Females	101	52 (34)	119	51 (63)	−13 (−54, 27)	0.506
**Sucrose (g)**						
Males	53	−4 (36)	78	32 (43)	−54 (−79, −29)	<0.001
Females	49	0 (23)	50	32 (31)	−34 (−57, −10)	0.006
**Fructose (g)**						
Males	22	29 (16)	23	21 (19)	8 (−3, 20)	0.139
Females	17	31 (7)	27	11 (15)	12 (0, 24)	0.044
**Glucose (g)**						
Males	22	22 (5)	23	20 (6)	3 (−4, 11)	0.538
Females	16	23 (3)	26	9 (5)	5 (−5, 15)	0.33
**Vitamin C (mg)**						
Males	106	−6 (64)	106	63 (269)	−68 (−212, 76)	0.339
Females	92	28 (61)	90	−25 (91)	55 (−85, 195)	0.426
**Dietary fiber (g)**						
Males	34	−2 (18)	28	−7 (11)	9 (1, 16)	0.004
Females	25	6 (5)	20	−3 (8)	12 (5, 19)	0.002
**Alcohol (g)**						
Males	13	−6 (10)	1	9 (11)	−11 (−23, 0)	0.054
Females	4	3 (12)	6	6 (13)	−4 (−14, 7)	0.459

a *ANCOVA was used to obtain estimate with adjustment for baseline values*.

There were no overall significant differences in the effects of treatment on cardiometabolic variables or body composition (Table 3). However, there was a significant sex by treatment interaction (*P* = 0.032) for serum uric acid. Amongst men uric acid was 57 μmol/L higher in those in the soft drink group (*P* = 0.008) but there was no effect in women. There was also a significant sex interaction (*P* = 0.034) for insulin sensitivity index. Insulin sensitivity declined amongst men and increased amongst women in the soft drink group compared with the fruit groups but the differences between treatments were not significant for men or for women. In a multivariate adjusted model examining the effect of treatment on serum uric acid soft drink treatment (*P* = 0.001), baseline BMI (*P* < 0.001) and increased alcohol intake (*P* = 0.032) were associated with higher uric acid while female sex (*P* = 0.002) was associated with lower uric acid (Table 4).

**Table 3:** Change in body composition and clinical measures from baseline to week 4 and difference between treatments.

	**Fruit**	**Soft drink**	**Treatment effect^b^**	***P*-value for difference**
	**Change from baseline^a^**	**Change from baseline^a^**		
**Plasma uric acid (μmol/L)**				
All participants	−6.00 (37.75)	4.37 (49.15)	14.09 (−17.77, 45.96)	0.375
Males	−14.67 (31.89)	22.80 (39.42)	57.17 (16.35, 98.00)^c^	0.008
Females	2.67 (42.92)	−16.11 (52.82)	−1.33 (−6.88, 9.53)^c^	0.295
			*P for sex interaction*	*0.032*
**Insulin sensitivity index^d^**				
All participants	−0.21 (0.99)	−0.27 (1.36)	−0.115 (−0.89, 0.66)	0.765
Males	−0.14 (1.24)	−1.01 (1.27)	−0.84 (−1.80, 0.13)^c^	0.088
Females	−0.28 (0.73)	0.55 (0.94)	0.68 (−0.32, 1.69)^c^	0.173
			*P for sex interaction*	*0.034*
Weight, kg	0.03 (1.02)	0.24 (1.67)	0.26 (−0.67, 1.18)	0.579
**Waist circumference, cm**				
All participants	0.72 (1.83)	1.48 (1.62)	0.79 (−0.33, 1.91)	0.161
Males	1.07 (1.43)	0.87 (1.58)	−0.12 (–1.68, 1.72)	0.863
Females	0.36 (2.18)	2.17 (1.46)	1.76 (0.17, 3.35)	0.031
			*P for sex interaction*	*0.093*
Body fat, %	−0.18 (1.06)	0.27 (1.28)	0.46 (−0.32, 1.24)	0.235
Systolic BP, mm Hg	2.17 (9.54)	0.63 (6.62)	−2.18 (−7.16, 2.80)	0.381
Diastolic BP, mm Hg	4.11 (11.50)	0.32 (8.33)	−4.96 (−10.84, 0.92)	0.272
Triglycerides, mmol/L	0.04 (0.36)	0.12 (0.10)	0.07 (−0.18, 0.32)	0.571
LDL-cholesterol, mmol/L	0.03 (0.36)	0.08 (0.42)	0.08 (−0.18, 0.34)	0.526
HDL-cholesterol, mmol/L	−0.04 (0.17)	−0.04 (0.15)	0.004 (−0.08, 0.09)	0.932
Total cholesterol, mmol/L	0.01 (0.41)	0.09 (0.53)	0.12 (−0.19, 0.43)	0.438
Plasma insulin, mIU/ml	0.02 (0.71)	1.94 (5.97)	1.54 (−2.48, 5.56)	0.443
Fasting glucose, mmol/L	0.02 (0.39)	0.02 (0.32)	−0.05 (−0.27, 0.17)	0.632
CRP, mg/L	0.74 (0.39)	0.21 (0.69)	0.10 (−1.54, 1.74)	0.902

a *Values are mean change (SD)*;

b *ANCOVA was used to obtain estimate with adjustment for baseline values with fruit as the reference group*;

c *ANCOVA was used to obtain estimate with adjustment for baseline uric acid and an interaction effect between treatment and sex*;

d *McAuley Insulin sensitivity index: Mffm/I = exp[2.63 – 0.28 ln(insulin) – 0.31 ln(TAG)]*.

**Table 4:** Multivariate analysis of covariance of the effect of treatment and confounding variables on uric acid at week 4.

	**β (SE)**	***P-*value**	***R*^**2**^**
**Model 1**		<0.0001	0.756
Soft drink	14.10 (15.70)	0.375	
**Model 2**		<0.0001	0.817
Soft drink	57.17 (20.04)	0.008	
Female	−20.61 (24.64)	0.409	
**Model 3**		<0.0001	0.871
Soft drink	65.50 (17.28)	0.001	
Female	−38.21 (21.63)	0.087	
Baseline BMI	5.20 (1.45)	0.001	
**Model 4**		<0.0001	0.893
Soft drink	58.3 (15.5)	0.001	
Female	−65.0 (19.5)	0.002	
Baseline BMI	6.7 (1.3)	<0.001	
Alcohol intake (g)	1.0 (0.5)	0.032	

*Model 1 covariate, baseline uric acid; Model 2 covariates, baseline uric acid, sex by group interaction term; Model 3 covariates, baseline uric acid, sex by group interaction term, baseline BMI; Model 4 covariates, baseline uric acid, sex by group interaction term, baseline BMI, change in alcohol intake during intervention period*.

## Discussion

We found that amongst overweight people increasing sugars intake either in the form of added sugars in soft drinks, or natural sugars from fruit did not lead to any deleterious changes in body weight or cardiometabolic risk factors and with no difference in effects between the two interventions. The absence of any change in weight despite being provided with additional energy from sugars (~1,800 kJ/d) in the form of fruit or soft drinks suggest that participants sub-consciously moderated their overall food intake to compensate. In a *post-hoc* analysis, we did find that consumption of soft drink resulted in a non-significant rise in plasma uric acid levels among men, while intake of an equivalent amount of fruit did not, with the difference between interventions being statistically significant. This research suggests that health promotion strategies to reduce the prevalence of non-communicable diseases must consider more than simply recommending the replacement of added sugars with fruit.

The finding that participants did not gain weight as a result being asked to consume additional sugar on a daily basis for 4 weeks was surprising. Many previous studies that have reported that participants gain weight when provided with sugar sweetened foods and drinks in addition to their usual diets, in an *ad libitum* context (2). We recruited participants that were willing to increase their sugars intake and were therefore likely to be relatively unconcerned about gaining weight. Evidence suggests that these type of participants may be more responsive to appetite and satiety cues than participants who are worried about gaining weight (27). However, if our study had been conducted over a longer period of time it is possible that we may have observed weight gain as our participants became habituated to a higher sugar intakes.

Our study has suggests that fructose intake from whole fruit may be handled differently by the body than added sugars from soft drinks leading to the observed rise in uric acid amongst men (7). However, it is also conceivable that differences in alcohol intake, which increased more in the soft drink group, might also explain the effect. Nevertheless fruit is important as fruit provides many beneficial dietary components, and those that would try to restrict fruit on the basis of its high fructose or sugars content would therefore reduce intake of these beneficial nutrients unnecessarily.

In this study the difference in plasma uric acid of 57 μmol/L between treatments amongst males is not only statistically significant, but is also large enough to be of clinical importance in the etiology of gout. While this was not a mechanistic study, potential reasons for the difference in uric acid between male groups should be explored. Firstly, it is not surprising that no difference in plasma uric acid was seen between female intervention groups, as high plasma uric acid levels and gout are characteristically more prevalent among men (28). During the intervention period, fiber intake was significantly higher among men and women consuming fruit (by 9–12 g/d), while total energy intake appeared higher (~1,000 kJ/d) among males consuming soft drinks. Thus, it is possible that fruit, due to its high fiber content, was more satiating and therefore conferred a reduced overall energy intake among men, leading to a lower plasma uric acid level. If continued longer, this difference in energy intake between male groups may have resulted in a significant difference in weight gain. The intrinsic fiber content of fruit may also have slowed the digestion rate of fructose, producing portal fructose concentrations that did not exceed the capacity of the small intestine and liver to metabolize fructose via routes other than those resulting in uric acid production (29–31). In addition, the fruit was consumed on average over 4 occasions per day (1.5 items/occasion) compared with 1.5 occasions for soft drink, further reducing the bolus dose of fructose consumed. When fructose is consumed in conjunction with glucose, as is the case in sugar-sweetened soft drinks, its absorption is enhanced (31) and it is unable to be metabolized down the glycogenic pathway, which is occupied by glucose (32).

A further possibility is that the higher vitamin C content of fruit reduced the effect of fructose on plasma uric acid levels. In a meta-analysis of 13 vitamin C supplementation studies reporting serum uric acid levels, a statistically significant mean reduction in serum uric acid of 21 μmol/L was observed with a median supplementary intake of 500 mg/d vitamin C (33). In this study, however, fruits with low vitamin C contents were chosen, and the difference in vitamin C intakes, although not significantly different, appeared higher in men receiving the soft drink treatment.

A strength of this study is that the amount of additional total sugars (~100 g/d) and fructose (~50 g/d) participants were required to consume was within the range consumed by populations, a factor often neglected in studies of this kind (34, 35). The average American consumes ~75 g/d of fructose (36) while the median usual daily intake of total sugars in NewZealand is ~120 g for males and 96 g for females (37). The fact that we did not observe the weight gain or other cardiometabolic effects observed in other studies (2, 3) suggests that public health approaches to reducing population obesity focusing only on reducing sugary drink intake may not be particularly effective and should focus on improving the quality of population diets as a whole.

Several factors limit the interpretation of the current study. While it is important to note that a meaningful difference in plasma uric acid in men was observed *without* evidence of a difference in weight gain, this study was underpowered to detect such a difference. In addition, it is possible that the intervention was not of sufficient duration to see an effect of soft drink consumption on other cardiometabolic risk factors also thought to be elevated by high fructose intakes and associated with hyperuricaemia (7, 31). A further, appropriately powered, longer-term study in overweight men is therefore warranted.

## Data Availability Statement

The original contributions presented in the study are included in the article/supplementary material, further inquiries can be directed to the corresponding author/s.

## Ethics Statement

This study was approved by University of Otago Human Ethics Committee (Ref: 12/197). The trial was registered with the Australian New Zealand Clinical Trials Registry: ACTRN12612000874819; http://www.anzctr.org.au. The patients/participants provided their written informed consent to participate in this study.

## Author Contributions

LT designed the research and had primary responsibility for the final content. SM conducted the research. LT, SM, and FO performed the statistical analysis and wrote the manuscript. All authors have read and approved the final manuscript.

## Conflict of Interest

The authors declare that the research was conducted in the absence of any commercial or financial relationships that could be construed as a potential conflict of interest.
